# Predicting wheat yield from 2001 to 2020 in Hebei Province at county and pixel levels based on synthesized time series images of Landsat and MODIS

**DOI:** 10.1038/s41598-024-67109-3

**Published:** 2024-07-13

**Authors:** Guanjin Zhang, Siti Nur Aliaa Binti Roslan, Helmi Zulhaidi Mohd Shafri, Yanxi Zhao, Ci Wang, Ling Quan

**Affiliations:** 1 Department of Civil Engineering, Faculty of Engineering, University Putra Malaysia, 43400 Serdang, Selangor Malaysia; 2 College of Resource and Environment, Anhui Science and Technology University, Chuzhou, 233100 China; 3 College of Agriculture, Nanjing Agricultural University, Nanjing, 210095 China; 4 School of Physics and Electronic-Electrical Engineering, Ningxia University, Yinchuan, 750021 China

**Keywords:** Hebei Province, Wheat, Synthesized images, Vegetation index, Deep learning, Plant sciences, Ecology

## Abstract

To obtain seasonable and precise crop yield information with fine resolution is very important for ensuring the food security. However, the quantity and quality of available images and the selection of prediction variables often limit the performance of yield prediction. In our study, the synthesized images of Landsat and MODIS were used to provide remote sensing (RS) variables, which can fill the missing values of Landsat images well and cover the study area completely. The deep learning (DL) was used to combine different vegetation index (VI) with climate data to build wheat yield prediction model in Hebei Province (HB). The results showed that kernel NDVI (kNDVI) and near-infrared reflectance (NIRv) slightly outperform normalized difference vegetation index (NDVI) in yield prediction. And the regression algorithm had a more prominent effect on yield prediction, while the yield prediction model using Long Short-Term Memory (LSTM) outperformed the yield prediction model using Light Gradient Boosting Machine (LGBM). The model combining LSTM algorithm and NIRv had the best prediction effect and relatively stable performance in single year. The optimal model was then used to generate 30 m resolution wheat yield maps in the past 20 years, with higher overall accuracy. In addition, we can define the optimum prediction time at April, which can consider simultaneously the performance and lead time. In general, we expect that this prediction model can provide important information to understand and ensure food security.

## Introduction

Food security is related to many important issues (e.g., the stability of society and economy), which is very important for the county^1,2^. Crop yield can affect the overall supply chain in the agricultural economy, which is closely related to food security^3,4^. Crop growth estimation and yield prediction are helpful to formulate reasonable management measures for farmers to ensure the stability of the grain market^5^. Wheat is the main grain crops in China, and the supply of wheat is vital to the stability of food market^6,7^. Therefore, to obtain timely and accurate wheat yield information is an important part of ensuring food security.

Surface observation method can obtain the most accurate crop yield information. However, the method is time-consuming and costly, and it is difficult to apply in the large level areas^8^. Satellite images can provide plenty of information about crop growth and yield in large area from the variables such as vegetation index (VI) and soil moisture (SM)^9,10^. The correlation of variables and crop yield can be used to build yield prediction model^11^. Among them, NDVI is the most widely used remote sensing (RS) variable^12,13^. It is important for the accuracy of yield prediction model to select the optimal RS variable^14^. The solar-induced chlorophyll fluorescence (SIF) is a reemission of energy from plants within the wavelengths ranging from 600 to 800 nm, which has been used as a proxy of photosynthesis^15^. The SIF data holds great potential in predicting crop yield^16^. The SIF data has higher accuracy in the prediction of crop yield as compared with NDVI and EVI^17^. Therefore, SIF products have been applied to predict crop yield in many studies^18,19^. However, the spatial resolution of most SIF products are generally coarse^20^. This limits the application of SIF images in obtaining crop yield information with fine spatial resolution. Badgley et al*.*^21^ found that near-infrared reflectance (NIRv) processed by theoretical derivations can be as the effective substitution of SIF. In some studies, NIRv has been used to assess crop yield^22,23^. On the other hand, there is stronger correlation between emerging kernel NDVI (kNDVI) and some independent products (e.g., SIF) than NDVI and NIRv^24^. Amin et al*.*^25^ found that kNDVI outperforms NDVI in prediction precision and timeliness. The selection of optimal RS variable is an issue that cannot be ignored in crop yield prediction. However, there have been no studies to compare the capacity of emerging VIs in the yield prediction.

Linear regression algorithm is the most generally used statistical algorithm for building yield prediction model^26,27^. However, considering the large spatial heterogeneity of meteorological conditions and management measures in large-level region, the respond of crop to external environment conditions is nonlinear^28–30^. This increases the uncertainty of yield prediction model. Compared to linear regression algorithm, machine learning (ML) can capture the nonlinear respond of crop to environment variables in yield prediction^31–33^. Deep learning (DL) model is the more advanced ML model that transform raw input data over stacked nonlinear layers to improve model performance^34–36^. Among them, long short-term memory (LSTM) has wide application and better performance in yield prediction researches^12,37,38^. However, ML and DL have a large demand for training samples, and it is costly to obtain enough data samples in the large region^7,39^. Many studies have used statistical yield data and ML algorithms to predict different crop yield at county level^40–42^. Nevertheless, the level inconsistency will affect the stability of the model when the model is used to crop yield prediction at pixel level^6,43^. Therefore, to develop a robust yield prediction model at multi-level is a challenging issue, especially in the dominant region of peasant economy.

Moreover, long-term high resolution crop yield data is helpful to exploring the effects of climate change on agricultural production^7,44^. The satellite images from Landsat can cover past decades. However, due to the limitations of revisit period and rainy weather conditions, it is difficult to obtain high-quality time series images from Landsat during crop growth period^45^. Sentinel-2 can only provide RS images with higher temporal and spatial resolution after 2015. On the other hand, MODIS can provide high temporal resolution satellite images after 2000, but with low spatial resolution. Therefore, the integration of satellite images from Landsat and MODIS has great potential in studying time series change of crop yield.

This study aims to: (1) reconstruct time series images with high spatial resolution by integrate Landsat and MODIS satellite images; (2) compare the effects of NDVI and emerging VIs in yield prediction; (3) develop yield prediction model to obtain robust prediction at county level and pixel level.

## Data and research methods

### Study area

**Figure 1:**
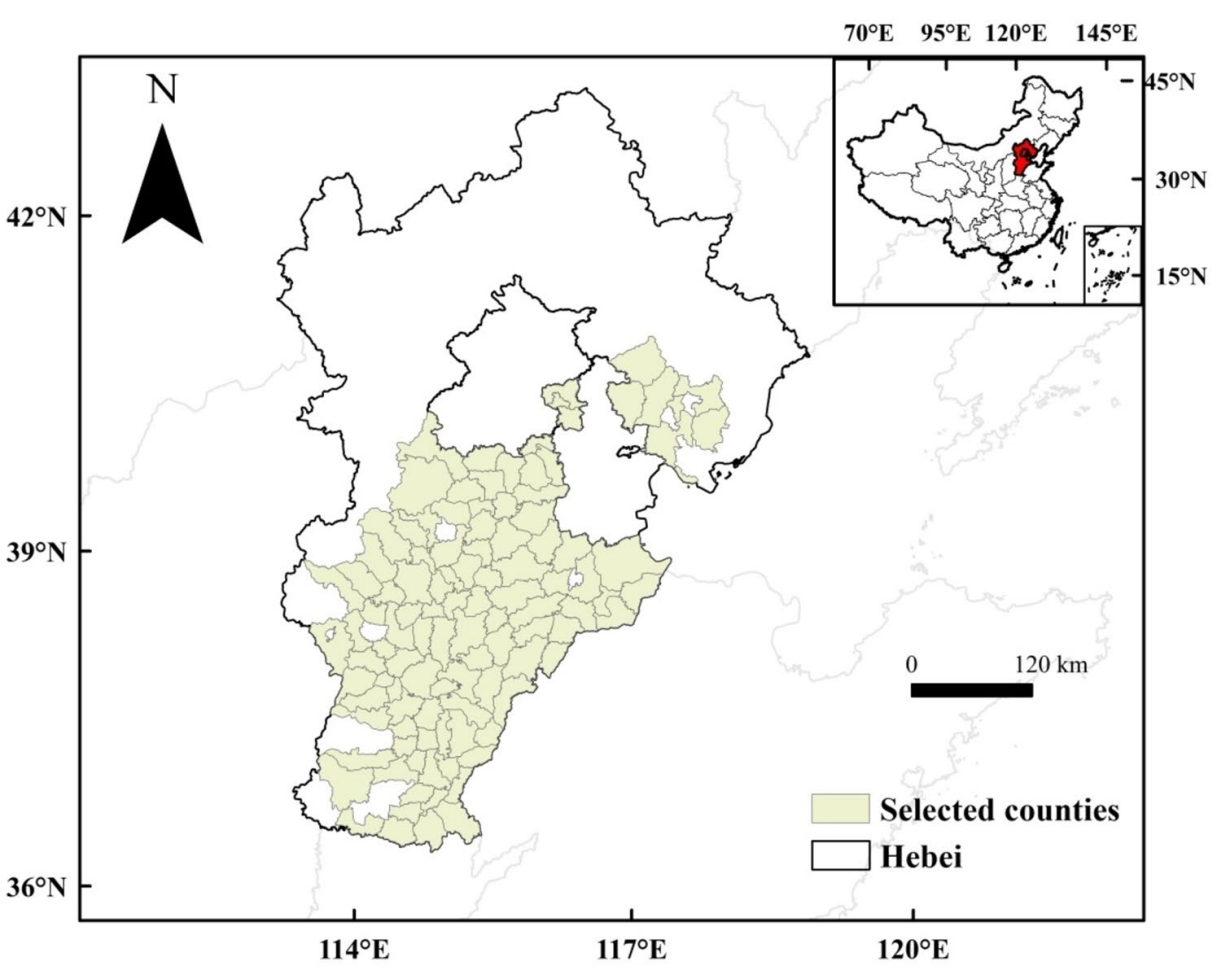
The study area and boundary of the selected counties in Hebei Province.

Hebei Province (HB) is located in North China Plain (NCP), which is the study area in our study (Fig. 1). The plain is concentrated in the southeast of Hebei Province, and the west and north of HB are mainly mountainous. The crops planted in the plain of HB include wheat, maize and cotton. The major cropping system is double-cropping system of winter wheat-summer maize, and wheat is usually planted in late September or early October and harvested in late May or early June^46^.

### Data

The data mainly include satellite data, climate data, statistics yield data at county level and observed yield data at site level.

#### Satellite data

In this study, we selected three VIs to build the yield prediction model, including NDVI, kNDVI and NIRv. We can extract the VIs during 2001–2020 from both Landsat and MOD09A1 products. Among them, the temporal and spatial resolution of Landsat images were 16 d and 30 m, while the temporal resolution and spatial resolution of MOD09A1 images were 8 d and 500 m. The formulas are as follows:

1 $$\text{NDVI}=(\text{NIR}-\text{RED})/(\text{NIR}+\text{RED}),$$

2 $$\text{NIRv}=\left(\text{NDVI}-0.08\right)*\text{NIR},$$

3 $$\text{kNDVI}=\text{tanh}\left({\text{NDVI}}^{2}\right),$$

where RED and NIR represent red band, near infrared band, respectively.

#### Climate data

The five climate variables was selected as auxiliary data to build the yield prediction model, including precipitation (Pr), maximum temperature (Tmax), minimum temperature (Tmin), vapor pressure deficit (VPD) and soil moisture (SM). The above climate data from 2001 to 2020 are obtained from TerraClimate dataset (the temporal resolution and spatial resolution were monthly and ~ 4 km)^47^, which can be download in the GEE platform. The dataset has the advantages of low error and high precision as compared to other climate datasets.

#### Yield data

The statistics yield data at county level for wheat can be gained from the China Agricultural Statistical Yearbook (CASY). More than 2000 yield data for 107 selected counties from 2001 to 2020 were applied to train and validate yield prediction models. The observed yield data at 13 agro-meteorological sites (including Luancheng, Huanghua and so on) during 2001–2010 was gained from China’s Meteorological Administration (CMA), which was used to validate the performance of yield prediction model at pixel level.

### Research methods

**Figure 2:**
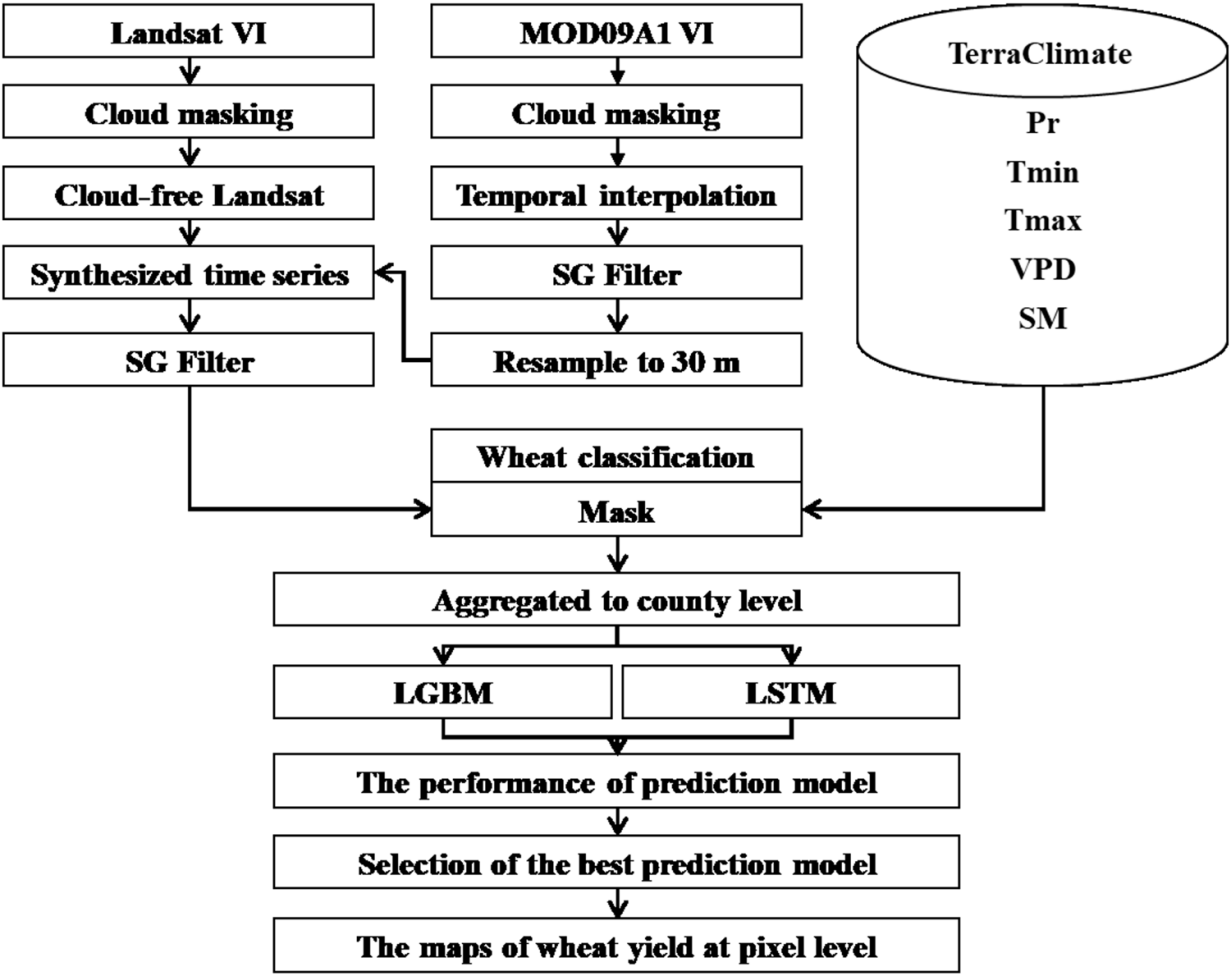
The architecture of data processing and yield prediction model.

The architecture of data processing and yield prediction model was shown in Fig. 2.

#### Data processing

**Figure 3:**
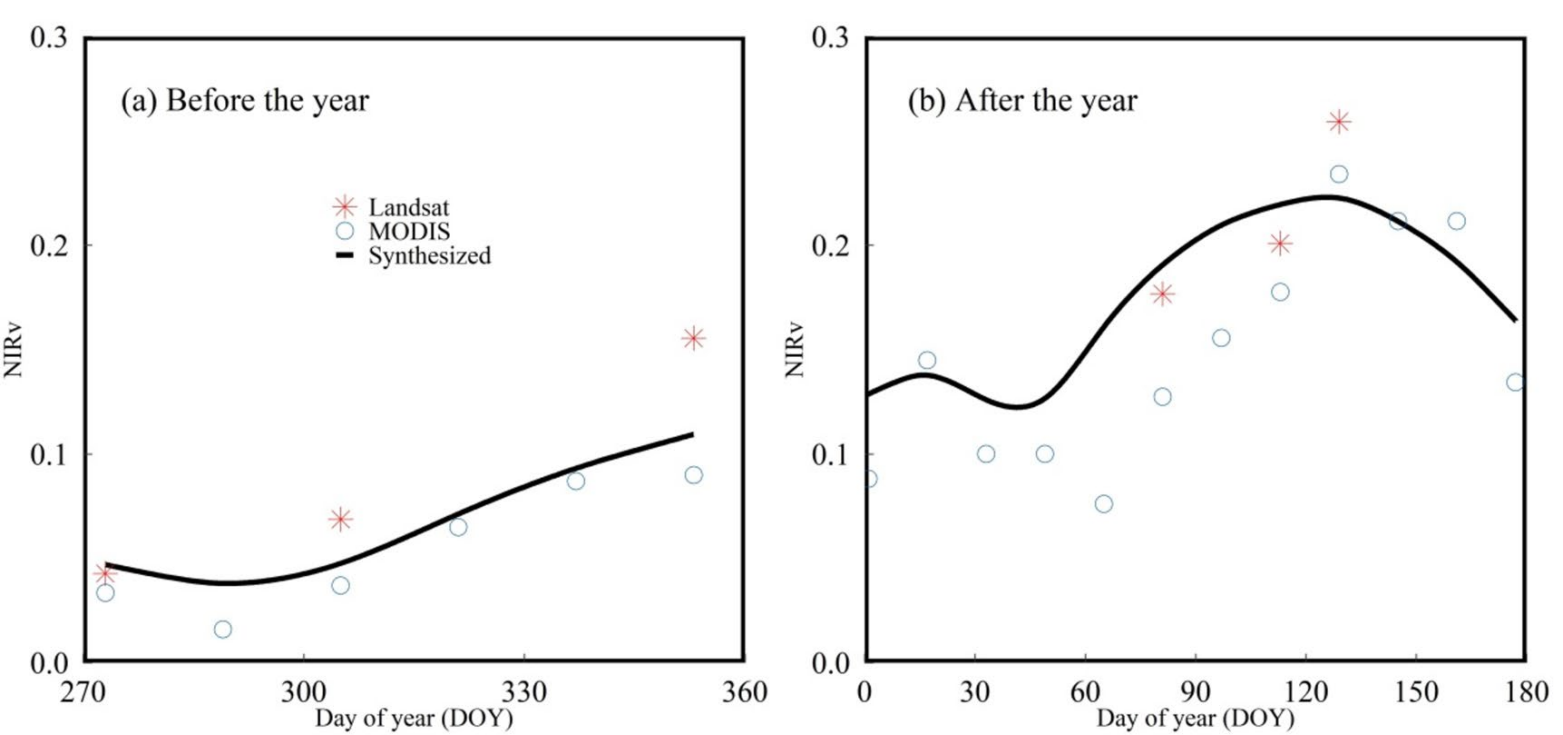
The synthesized NIRv time series curve of Landsat and MODIS after reconstruction for a pixel in Hebei Province, 2020.

**Figure 4:**
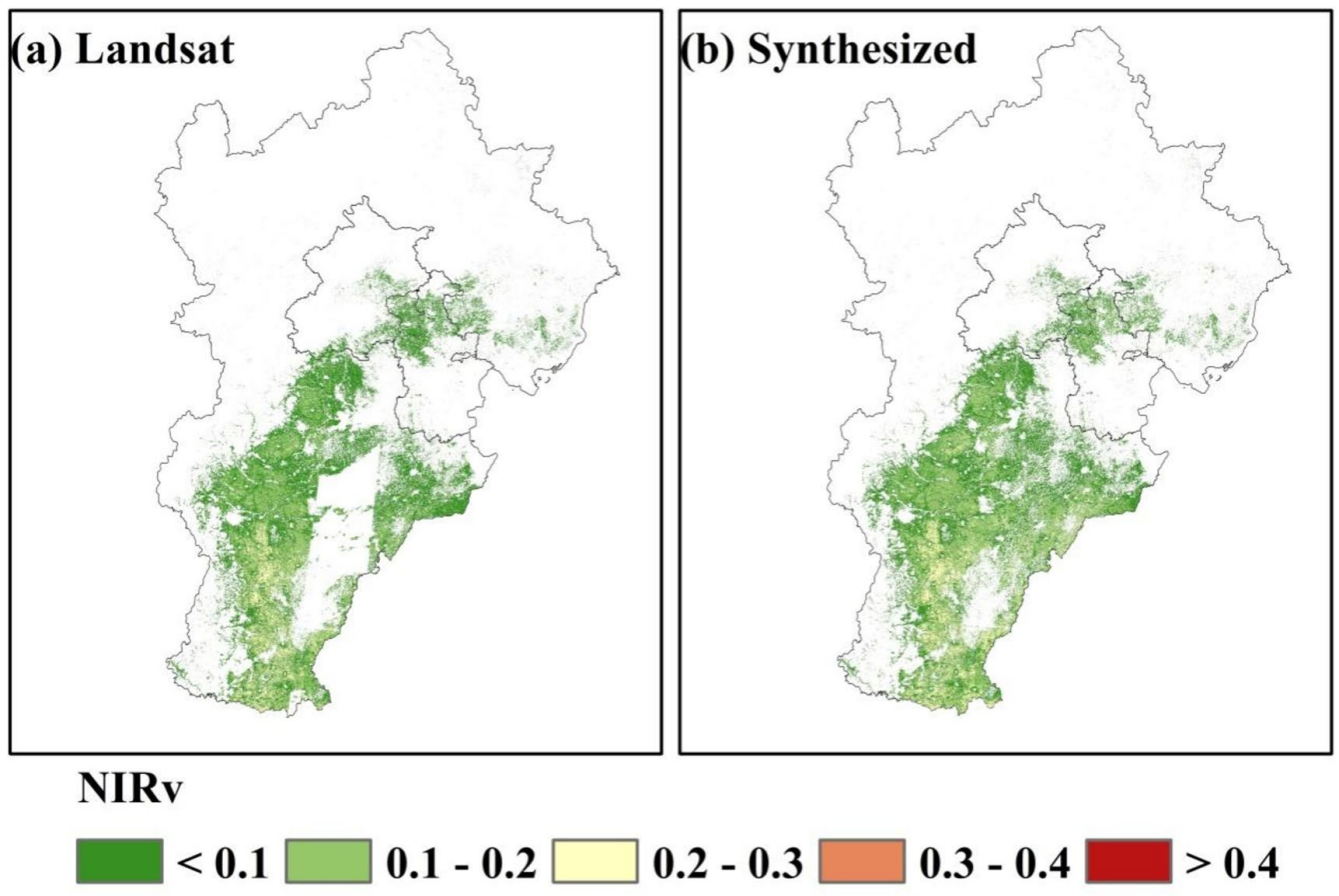
Comparison of original NIRv images from Landsat and the synthesized NIRv images of Landsat and MODIS after reconstruction in Hebei Province on March 5, 2020.

First, we calculated VIs from Landsat images. Then we obtained cloud-free satellite images by cloud masking. These cloud-free images were aggregate as monthly intervals (from October to the following May) using the maximum synthesis method. However, because of the low temporal resolution and the influence of cloudy and rainy weather condition, it is difficult for the cloud-free satellite images to continuously cover the study area. MODIS image products have high temporal resolution, and some studies have shown that MODIS products can be used as effective auxiliary data to reconstruct high-quality Landsat data^45^. In this study, we selected MOD09A1 images as auxiliary data. We generated MODIS cloud-free satellite images by using cloud masking. The temporal interpolation was used to fill the missing values in the MODIS images. The cloud-free MODIS images were aggregate as monthly intervals (from October to the following May) like Landsat. Savitzky-Golay (SG) filter was used to smooth the time series images to reduce the effect of noise. To fill gaps of Landsat images, MODIS images were resampled to 30 m spatial resolution based on the bilinear resampling method. Finally, the synthesized time series images was generated and the SG filter was used to smooth the time series. The synthesized NIRv time series curve of Landsat and MODIS after reconstruction was shown in Fig. 3. The synthesized images can fill the missing values of Landsat images well and cover Hebei Province completely (Fig. 4).

On the other hand, the monthly climate data was resampled to 30 m resolution. The satellite images and climate data were masked based on wheat classification result, which can be obtained from the study of Zhao et al*.*^7^. Finally, all input data were aggregated to a mean for each county after being masked by wheat planting areas.

#### Yield prediction model

In this study, satellite images, climate data and statistics yield data at county level were applied to build yield prediction model. Among them, statistics yield data was defined as the target variable in yield prediction model, while other data was set as predictive variable. All of the data samples were randomly split into 70% for training and 30% for validation. The ten-fold cross validation was used to optimize the parameters, and wheat yield prediction model was built by using input parameters with the best effect. To study the performance of selected VIs in the yield prediction model, this study set three sets of data input combinations for comparison, namely: (1) NDVI combined with climate data (NDVI); (2) NIRv combined with climate data (NIRv); (3) kNDVI combined with climate data (kNDVI).

LSTM algorithm is essentially specific form of Recurrent Neural Network (RNN)^48^. LSTM can solve the short-term memory problem of RNN by adding Gates, which makes RNN can effectively make use of the time series information. Many studies have shown that LSTM model has a good performance in crop yield prediction^12,18^. Furthermore, LGBM^49,50^ was defined as the benchmark model to compare with LSTM. LGBM is an improved algorithm using the traditional Gradient Boosting Decision Tree (GBDT). As compared with Extreme Gradient Boosting, LGBM has faster speed, higher computing efficiency and greater performance.

The study compared the influence of different VIs and regression methods in the yield prediction model. The root mean square error (RMSE) and coefficient of determination (R^2^) between predicted and statistical data were used to evaluate performance. We selected the optimal prediction model after the comparison. Then “leave one year out” experiment was implemented to assess the performance of the optimal model in each year. The “leave one year out” experiment is that the data of single year is used as test dataset, and the data of other years is used as training dataset. The RMSE and R^2^ between predicted and statistical data in single year were applied to evaluate performance of the optimal model. In addition, the relative error (RE) between predicted and statistical data in single year was used to study spatial distribution of prediction errors. Moreover, the optimal model was applied to generate wheat yield maps in HB during 2001–2020 at pixel level, while the RMSE and R^2^ were between predicted and observed yield (including statistical data and observed yield data at site level) calculated to assess the accuracy of prediction model at pixel level. Finally, we investigated the contribution of time series data during different growth periods for yield prediction model and analysed the optimum prediction time.

## Results

### Model performances

**Figure 5:**
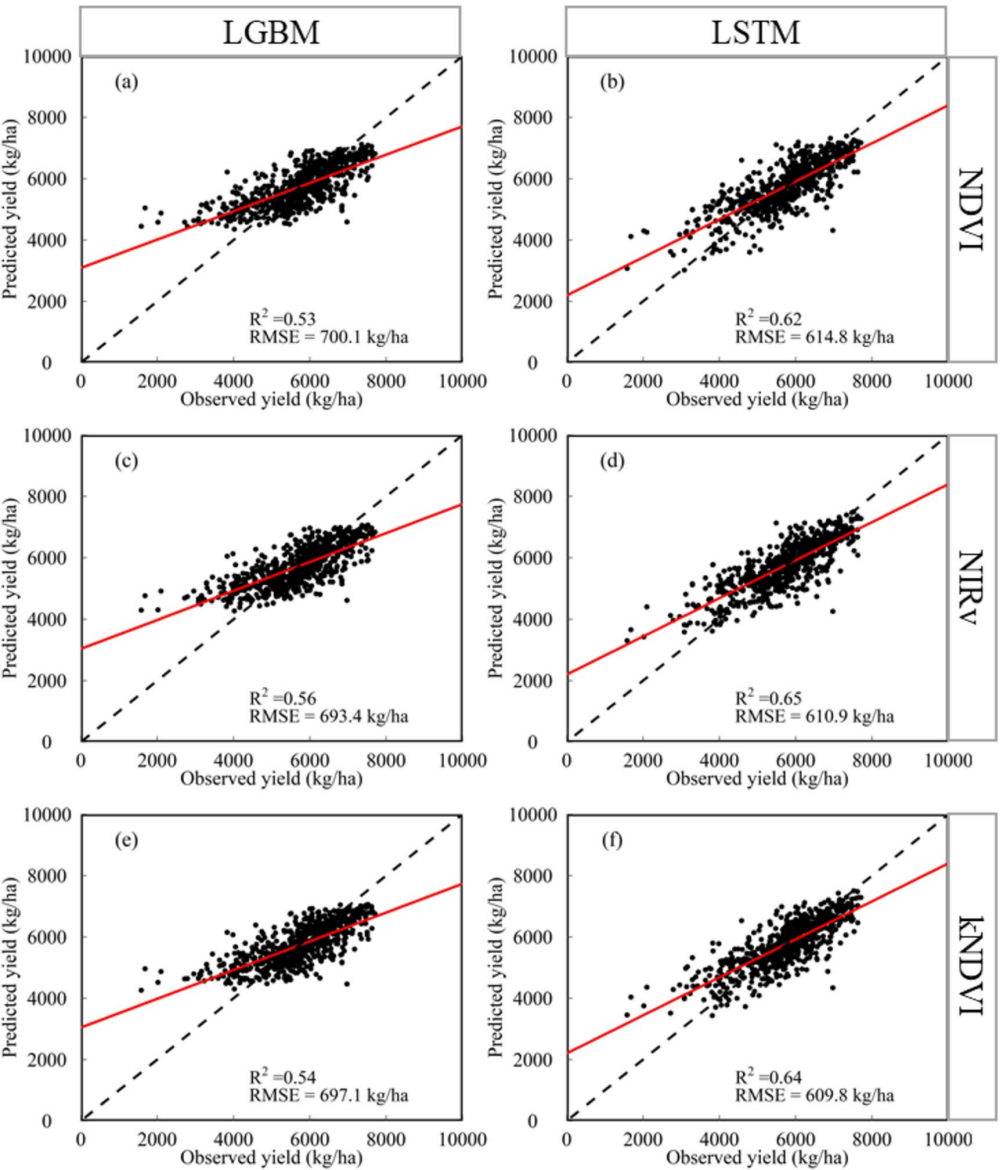
Performances of yield prediction model developed using different combination of input variables and regression algorithms based on random splitting validation (7:3) for the yield data during 2001–2020.

Performances of yield prediction model developed using different combination of input variables and regression algorithms using the yield data during 2001–2020 were shown in Fig. 5. Compared with NDVI (R^2^ was between 0.53 and 0.62, RMSE was between 614.8 and 700.1 kg/ha), the effect of the emerging VIs (NIRv and kNDVI) was slightly better (NIRv: R^2^ was between 0.56 and 0.65, RMSE was between 610.9 and 693.4 kg/ha; kNDVI: R^2^ was between 0.54 and 0.64, RMSE was between 609.8 and 697.1 kg/ha). The improvement was not significant. Meanwhile, NIRv and kNDVI have similar performances in yield prediction. On the other hand, the optimal selection of regression model was more significant for the improvement of yield prediction model than selection of VIs. The yield prediction model using LSTM algorithm outperformed the model using LGBM (LSTM: R^2^ was between 0.62 and 0.65, RMSE was between 609.8 and 614.8 kg/ha; LGBM: R^2^ was between 0.53 and 0.56, RMSE was between 693.4 and 700.1 kg/ha). Therefore, the yield prediction model built by combining NIRv and LSTM algorithm was defined as the optimal model, which was used for further study.

### Performance of the optimal model

**Figure 6:**
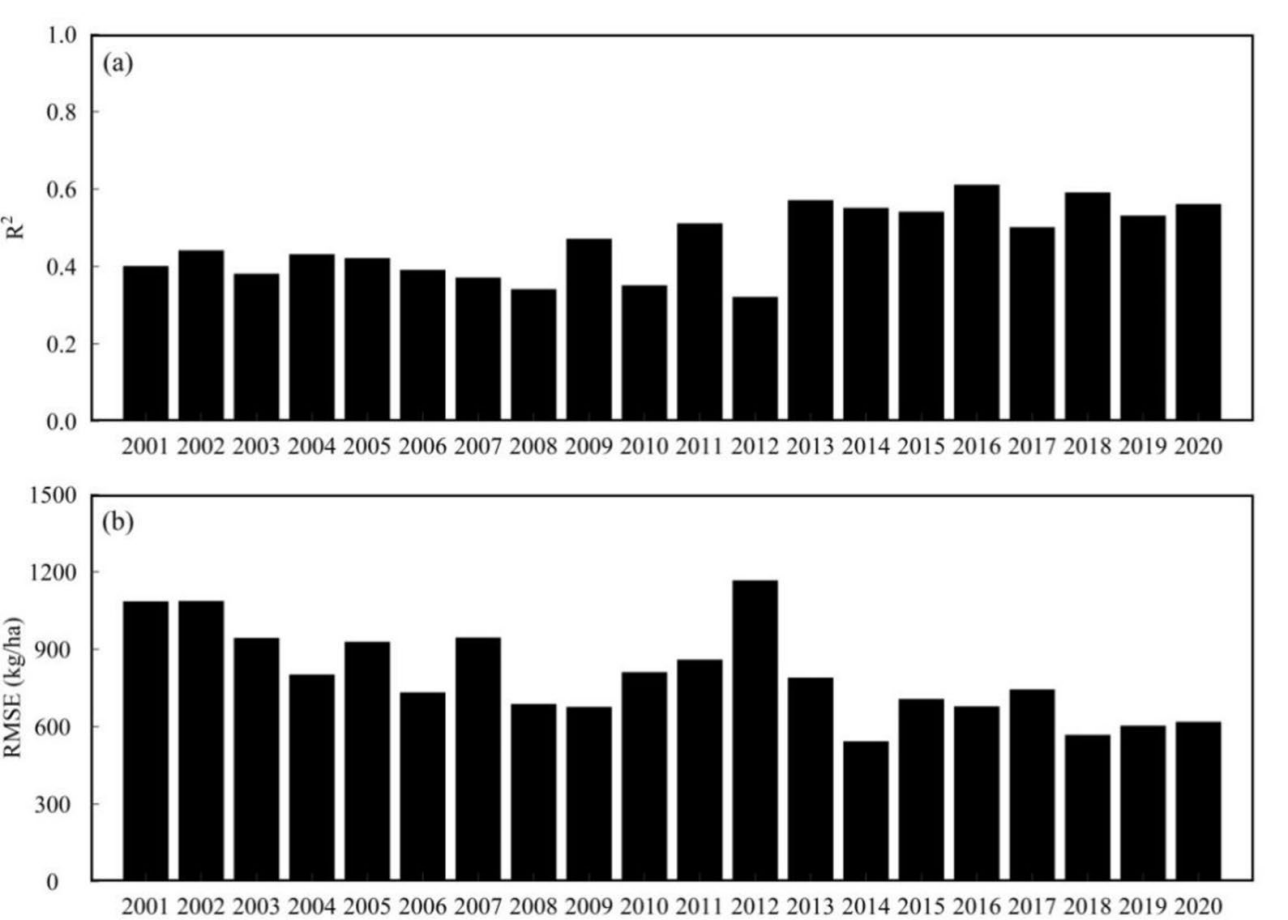
Comparisons of the predicted yield using the optimal model and statistical data in HB during 2001–2020.

Moreover, we evaluated performance of the optimal model for yield prediction in single year using “leave one year out”. The comparisons of predicted yield based on the optimal model and statistical data in HB during 2001–2020 were shown in Fig. 6. In general, the R^2^ values in the single year were between 0.32 and 0.61, while the RMSE values in the single year were between 541.3 and 1165 kg/ha. Taking year 2012 as the dividing line, the performance of the optimal model after 2012 (R^2^ was from 0.5 to 0.61, RMSE was from 541.3 to 787.6 kg/ha) was relatively better than the performance of the optimal model before 2012 (R^2^ was from 0.32 to 0.51, RMSE was from 674.2 to 1165 kg/ha). This may be because of the limitation for the quantity and quality of early cloud-free images from Landsat.

**Figure 7:**
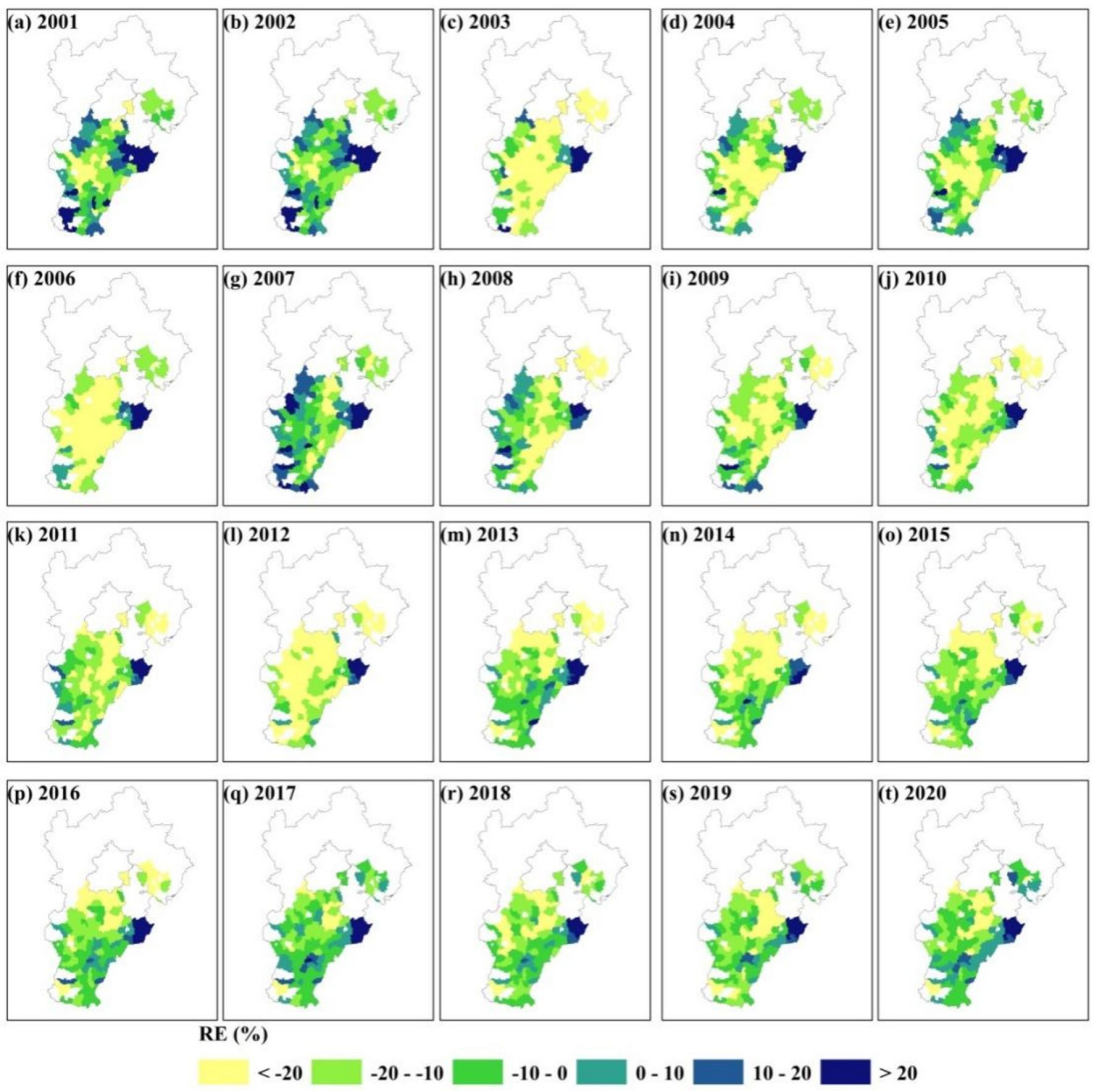
Spatial distribution pattern of RE between predicted yield using the optimal model and statistical data in HB during 2001–2020.

In addition, the RE index was used to study the spatial distribution pattern of uncertainty in yield prediction using the optimal model. The spatial distribution pattern of RE values between predicted yield using the optimal model and statistical data during 2001–2020 was shown in Fig. 7. We can find that there is an obvious overestimation (RE > 20%) in the northeast of Hebei Province during 2001–2020. This area is mainly rainfed agriculture, while other areas are irrigated agriculture. The uneven distribution of training samples may be the main reason. On the other hand, there is widespread underestimation in the central region of Hebei Province for some years prior to 2012. However, RE values between predicted yield and statistical data in most regions after 2012 roughly ranged from −20 to 20%.

### Wheat yield maps during 2001–2020 in HB

**Figure 8:**
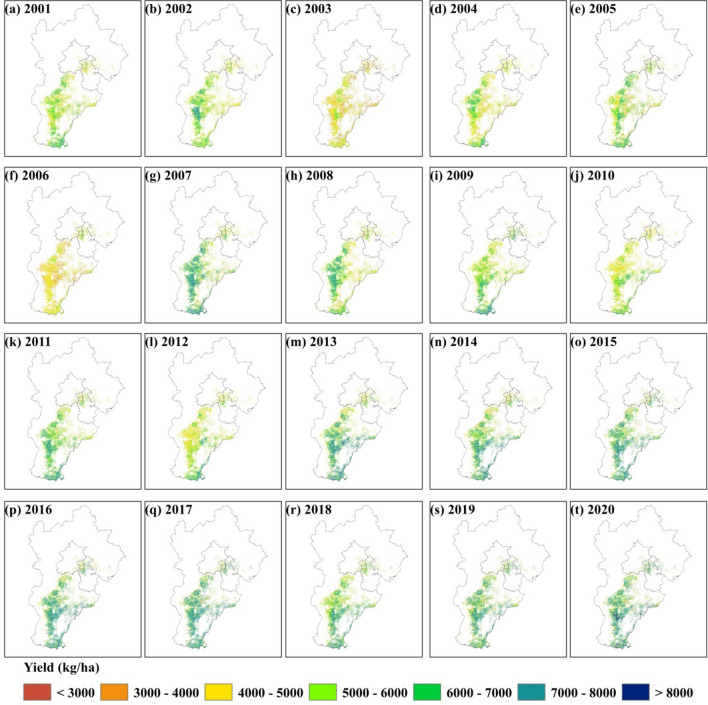
Spatial distribution pattern of predicted yield at pixel level based on the optimal model in HB during 2001–2020.

The optimal model was used to generate 30 m resolution yield maps at during 2001–2020 in HB (Fig. 8). Wheat yield maps revealed the heterogeneity in the different regions of HB. Wheat yield was higher in the central and southern regions of HB where wheat planting area was more concentrated, while the yield was lower in the northeastern regions where wheat planting area was more dispersed. This is consistent with the spatial distribution pattern of statistical data. In addition, we can find the increase of wheat yield in HB during the past 20 years, especially in the central-south of HB.

**Figure 9:**
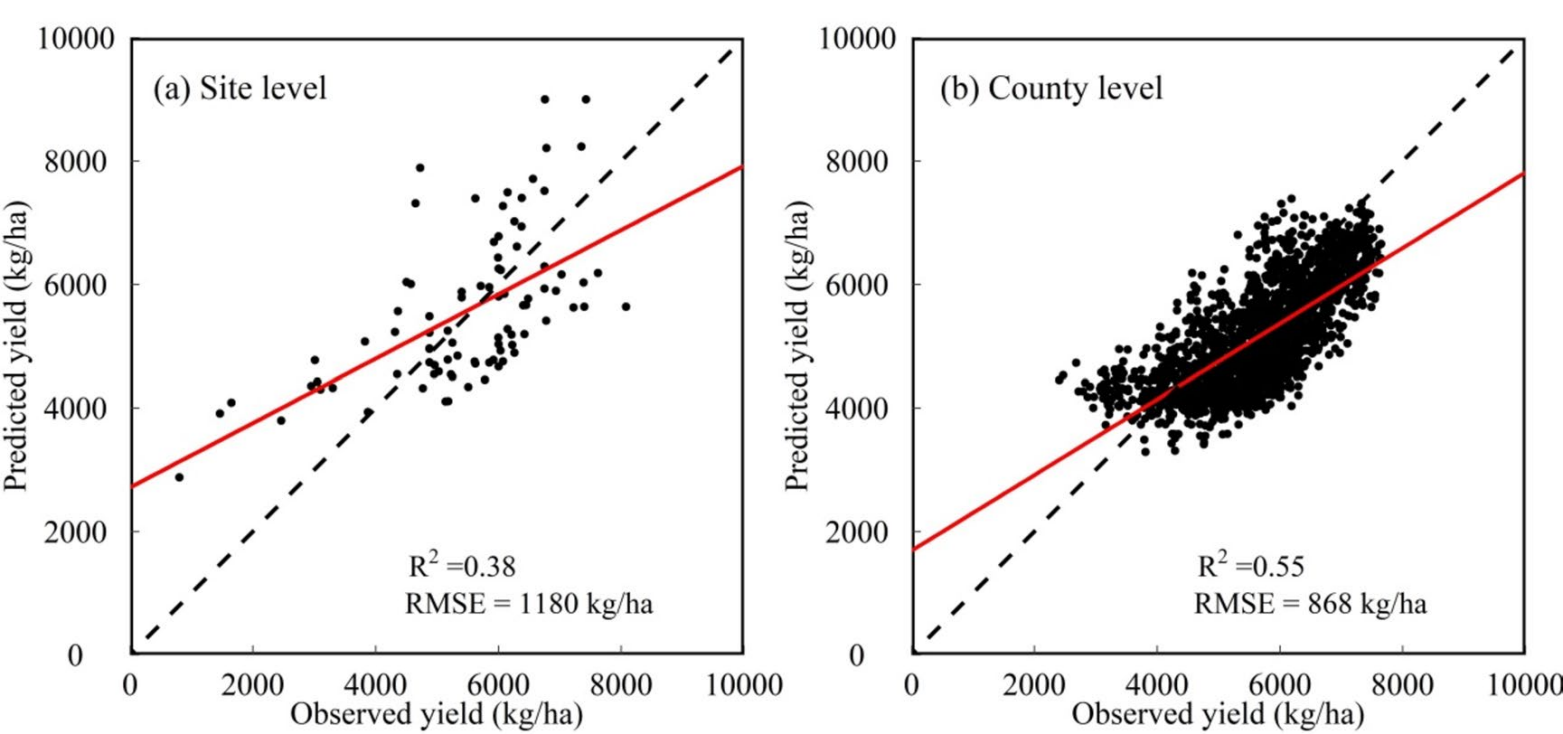
Performances of wheat yield maps at pixel level based on the optimal model compared with observed data at site level during 2001–2010 (**a**) and statistical data at county level during 2001–2020 (**b**).

Furthermore, we used statistics yield data at county level and observed data at site level to assess the quality of wheat yield maps with 30 m spatial resolution (Fig. 9). Compared with the reference data at site level, the yield maps had an overall an R^2^ of 0.38 and RMSE of 1180 kg/ha (Fig. 9a). Furthermore, we used the statistical data at county level to assess the yield maps aggregated to county level. Compared to the statistical data at county level, the yield maps had an overall an R^2^ of 0.55 and RMSE of 868 kg/ha (Fig. 9b). In general, the performance in yield prediction at county level using the optimal model was better than that in yield prediction at pixel level. This indicated that the training samples at different levels and regression algorithms will appear precision loss to some extent when the yield prediction was in the multi-level application.

### The optimum prediction time

**Figure 10:**
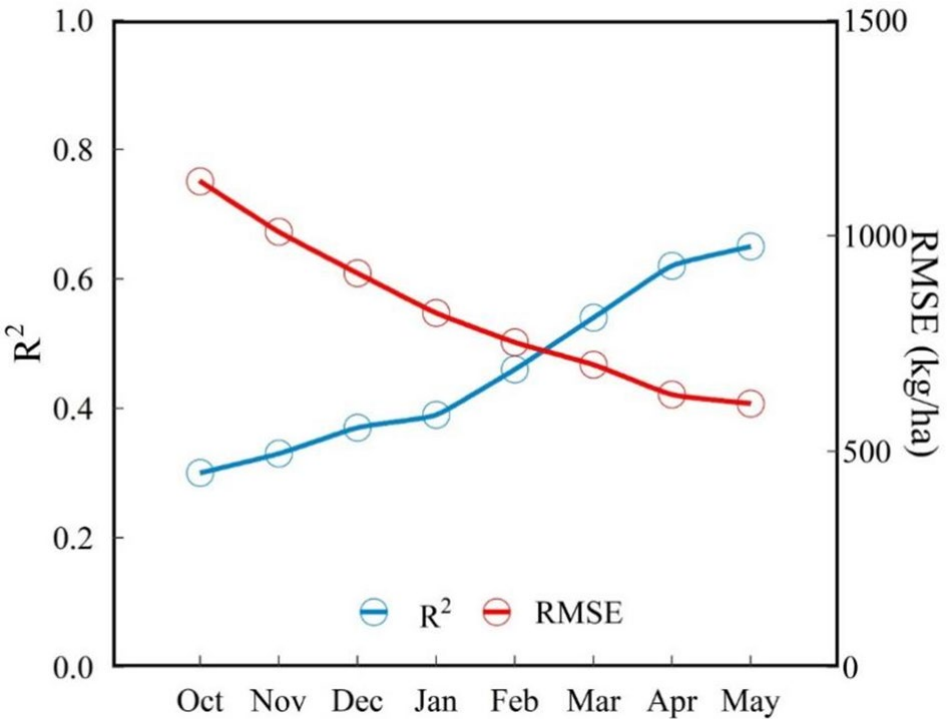
Model performance for predicted yield using forward month-based observations based on the optimal model.

The yield prediction model must measure the accuracy and prediction time. The performance for predicted yield using forward month-based data based on the optimal model was shown in Fig. 10. In this study, we found that the model accuracy increased as wheat growth period approached harvest time, especially after February. The increase of growth information reflected by VIs was conducive to the improvement of yield prediction model due to the acceleration of wheat growth after February. However, the growth rate of model accuracy slowed down after May as wheat approached maturity. The yield prediction in April can take into account the accuracy and advance prediction time, which was the optimum prediction time.

## Discussion

Landsat satellites can provide the most abundant public free historical RS images with 30 m resolution^45^, which has great potential for long time-series crop yield mapping. However, 16 d temporal resolution and the cloud-rain weather can limit the usage of Landsat images in large areas^7^. In our study, the missing values of Landsat images were filled by using MODIS series products with coarse spatial resolution, which have been smoothed and resampled. Then SG filter was used to remove the noise of synthesized images to reconstruct time series curve. The synthesized images can effectively fill in the missing value of original images from Landsat, and the study area can be completely covered. Furthermore, the synthetic images were applied to the generation of wheat yield maps with long time series in HB, and the overall accuracy of the yield maps was high. In addition, the accuracy of synthetic images can be further improved with the further development of data fusion method.

NDVI is the most widely used RS variable in yield prediction^12,13^. However, NDVI has a saturation effect when the green biomass is high. As a result, many improved VIs have been developed. As compared with NDVI, the emerging kNDVI and NIRv showed better performance in evaluating crop traits, phenology and yield^24,25^. In this study, the performance of yield prediction model combined with the emerging VIs has improved compared with that based on NDVI, but this improvement was not prominent. The additional input variables such as SIF products or extreme climate index data can be incorporated in yield prediction model to improve the accuracy of the models in some studies^41,51^. However, the spatial resolution of the additional data was usually lower, which was not convincing when the data was used to obtain crop yield information with high spatial resolution^52^. The development of more effective RS variables and auxiliary data with higher spatial resolution has great potential in the future study of yield prediction.

Relatively speaking, the regression algorithms have more prominent influence on the performance of yield prediction model as compared with the input variables. ML algorithms can capture the nonlinear respond of crop to climate and environment conditions, which outperformed traditional linear regression algorithms in yield prediction^8,53^. Deep learning algorithms can effectively process complicated time series data^54,55^. The accuracy of yield prediction model based on deep learning algorithms was better and more stable than that based on ML algorithms^41,56^. In our study, the accuracy of yield prediction model based on LSTM algorithm and NIRv was significantly higher than that using LGBM algorithm and NIRv. Meanwhile, the optimal model also has stable performance in predicting wheat yield for single year. Considering that deep learning algorithms have a large demand for data samples^57^, obtaining more data samples can improve the performance of deep learning algorithms further.

The farmers usually cultivated crops on small plots in the peasant economy. Due to the constraints of technology and funds, the production level of the peasant economy was relatively low, and the ability to resist natural disasters was weak^58^. Therefore, the spatial heterogeneity of crop growth and yield in the peasant economy dominant regions was large^59^. This increased the uncertainty of yield prediction on both temporal and spatial levels. Hebei Province was a traditional peasant economy dominant regions, and the spatial differences of management measures were obvious^17^. The northeastern region of Hebei Province was rainfed agriculture, while other areas were typically irrigated^7^. In our study, the optimal model significantly overestimated wheat yield in the rainfed agricultural regions. The data samples of irrigation county occupied a relatively large proportion in the total sample dataset, while the uneven distribution of input data may be the main reason for the error. In the next study, we can improve the robustness of the yield prediction model by optimizing the input dataset^39^.

## Conclusion

We used LSTM algorithm to integrate VIs and climate data to map wheat yield with 30 m resolution in HB during 2001–2020. The synthesized images of Landsat and MODIS were used to provide RS variables for yield prediction model. The performance of yield prediction model combined with the emerging VIs has improved slightly compared with that using NDVI. The optimal selection of regression model was more significant for the promotion of yield prediction model than the selection of VIs. The yield prediction model built by combining NIRv and LSTM algorithm was defined as the optimal model, which performed well in single year. The optimal model was applied to generate 30 m resolution wheat yield maps at during 2001–2020, and the yield maps had a higher overall accuracy. In addition, April was the optimum prediction time, which can consider simultaneously the precision and lead time. The multi-level wheat yield prediction framework provided in our study has great prospects for practical applications in the peasant economy dominant regions.

## Data Availability

The experimental research and pixel studies on plants (either cultivated or wild) were in accordance with relevant institutional, national, and international guidelines and legislation. The statistics yield data at county level for wheat can be gained from the China Agricultural Statistical Yearbook (CASY). The observed yield data at site level from 2001 to 2010 was gained from China’s Meteorological Administration (CMA). The datasets generated and analysed during the current study available from the corresponding author on reasonable request.
